# Mechanisms of Biocontrol on Poplar Anthracnose and Growth Promotion by *Trichoderma asperelloides* Tas369 and Tri-*Trichoderma* Composite Strain

**DOI:** 10.3390/microorganisms14081817

**Published:** 2026-08-18

**Authors:** Ning Kong, Bin Liu, Jing Han, Yongfeng Yang, Jia Yu, Dongyi Wang, Dechen Li, Zhihua Liu

**Affiliations:** 1 College of Land and Environment, Shenyang Agricultural University, Shenyang 110866, China; liubinwy@yeah.net (B.L.); hj0915@stu.syau.edu.cn (J.H.); 15040431422@163.com (D.L.); 2 College of Forestry, Shenyang Agricultural University, Shenyang 110866, China; kn96123@163.com (N.K.); yyongfeng96@163.com (Y.Y.); yujia199705@163.com (J.Y.); 18004126190@163.com (D.W.)

**Keywords:** *Trichoderma*, poplar anthracnose, growth promotion, biocontrol mechanism, stomatal density

## Abstract

To address the need for effective biocontrol of poplar anthracnose caused by *Colletotrichum gloeosporioides*, in this study, 20 *Trichoderma* strains were isolated from the rhizosphere of seven tree species on Tianzhu Mountain to screen for potent growth-promoting and antifungal agents. The inhibitory effects of the strains on *C. gloeosporioides* (Cgl) were evaluated through mycelial growth assays, volatile organic compound tests, and fermentation filtrate analysis, while their growth-promoting capabilities and induction of systemic resistance were assessed in poplar seedlings via biomass measurement, stomatal analysis, and gene expression profiling. The results demonstrated that *T. asperelloides* Tas369 significantly inhibited pathogen growth and improved seedling growth, increasing stomatal density by 134% and inducing phenylalanine ammonia-lyase (PAL) and peroxidase (POD) enzyme activities. Notably, *Trichoderma* composite (Tcom) outperformed single-strain treatments, achieving 89.8% control efficacy, reducing the malondialdehyde content by 1.1 mmol/g, and significantly upregulating the key defense genes *NPR1* (14.32-fold) and *PR1-1* (219.79-fold) through increased activity of the salicylic and jasmonic acid signalling pathways. In conclusion, while Tas369 exhibits strong individual potential, the Tcom consortium provides superior biocontrol through multiple mechanistic interactions, offering an effective and sustainable approach for managing poplar anthracnose in forestry applications.

## 1. Introduction

*Trichoderma* is widely recognized as a versatile biocontrol agent with dual functions: inhibiting pathogenic microorganisms and promoting plant growth [1]. When present in the rhizosphere, *Trichoderma* perceives root exudates to recognize potential host plants, then grows toward root tissues to colonize the rhizosphere or establish endophytic associations within plant tissues. This interaction activates endogenous signalling mechanisms in plants, regulating a variety of physiological processes [2], thereby promoting leaf growth and root branching, increasing plant vigor and stress resistance, and activating systemic resistance [3,4,5]. Owing to its excellent ecological adaptability, *Trichoderma* has emerged as an ideal candidate for the biological control of forest diseases [6].

The biocontrol mechanisms of *Trichoderma* against fungal diseases in plants have been extensively investigated. They can activate the innate defense systems of plants and exert their biocontrol effects through various bioactive substances and gene regulatory pathways [7]. For instance, heat shock proteins and hydrophobic proteins secreted by *Trichoderma* are involved in the activation of poplar defense systems, helping host trees adapt to environmental stresses and triggering defense responses [8]. Additionally, specific xylanases secreted by *Trichoderma* act as elicitors, inducing poplar to mount defense responses against *Beauveria bassiana* [9]. In the control of poplar leaf diseases, *Trichoderma* can also activate the SA and JA signalling pathways by upregulating the expression of pathogen-related (PR) protein genes and *MYC* genes (10.05-fold), thereby increasing poplar resistance to leaf blight [10,11,12]. These studies highlight the multifaceted mechanisms of *Trichoderma* in forest disease control. However, it is noteworthy that existing research focuses primarily on diseases such as leaf blight, while targeted studies on poplar anthracnose remain insufficient.

Poplar (*Populus* spp.) is a critically important fast-growing timber species characterized by rapid growth, ease of propagation, and strong environmental adaptability. It is widely utilized in industrial timber processing, bioenergy production, and the construction of ecological shelterbelts [13]. However, poplar anthracnose, primarily caused by the hemibiotrophic fungus *Colletotrichum gloeosporioides*, represents a devastating foliar disease that poses a major threat to poplar growth and productivity. This pathogen has a global distribution and has a broad host range, infecting numerous agricultural crops, ornamental plants, and forest trees, thereby causing substantial economic losses to both the forestry and agricultural sectors. Following infection, typical disease symptoms initially manifest as small brown spots on leaves and young shoots, which subsequently enlarge into conspicuous lesions characterized by grayish-white necrotic centers surrounded by purple-brown margins, ultimately impairing photosynthetic efficiency and overall plant vigor [14]. In severe cases, this disease leads to leaf scorching, premature defoliation, and shoot death, significantly inhibiting the growth of young trees and endangering the survival rate and vigor of poplar seedling stands [15]. Currently, research on the biological control of this disease is relatively limited. To date, only a few biocontrol bacteria-including *Bacillus subtilis* ZSH-1, *B. atrophaeus* XW-2, and *Pseudomonas atacamensis* GZ-3-have been reported to exhibit inhibitory activity against the pathogen, with the highest inhibition rate reaching 89.1% [16,17,18]. Therefore, given the multifaceted biocontrol potential of *Trichoderma* in poplar disease management and the current paucity of targeted research on poplar anthracnose biocontrol, systematic screening and investigations of the control functions of *Trichoderma* strains and their composite consortia are expected to reveal synergistic or complementary relationships with existing bacterial biocontrol resources. This, in turn, will aid in the development of biocontrol strategies for the sustainable management of poplar anthracnose.

In this study, *Trichoderma* strains were isolated from rhizosphere soils of seven tree species on Tianzhu Mountain, Liaoning, China, and were identified by morphological and molecular methods. On the basis of the results of competitive, antagonistic, and growth-promoting assays, a promising strain combination with significant biocontrol efficacy against poplar anthracnose and growth-promoting effects was selected. To explore the underlying mechanisms, key physiological and biochemical indicators—including antioxidant enzyme activities (MDA, PAL, POD), defense-related gene expression, and defensive cell morphology—were systematically analysed. Following a strategy integrating strain screening, functional validation, and mechanistic investigation, elite *Trichoderma* strains with both disease-suppressive and growth-promoting traits were obtained, and their biocontrol mechanisms were preliminarily elucidated. To further increase the biocontrol stability and spectrum, a *Trichoderma*-composite (Tcom) was developed by leveraging complementary antifungal actions and synergistic induction of plant defense pathways, aiming to establish an efficient and comprehensive green control strategy for poplar anthracnose and provide technical support for forestry applications.

## 2. Materials and Methods

### 2.1. Test Materials

Rhizosphere soil samples were collected from seven tree species growing on Tianzhu Mountain, Shenyang, China (E 123°37′53″, N 41°51′36″). For each tree species, two replicate subsamples were collected at depths of 10–20 cm. All the samples were placed in sterile self-sealing plastic bags, transported to the laboratory in an ice box, and stored at 4 °C. The pathogenic fungus employed in this experiment was *C. gloeosporioides* (Cgl), which was preserved on PDA slants at 4 °C and stored in 20% glycerol for a long period of time at −80 °C in the Laboratory of Biocontrol Mechanisms, College of Forestry, Shenyang Agricultural University. Uniform three-month-old potted *Populus davidiana* × *P. alba* var. *pyramidalis* (PdPap) seedlings with consistent heights and ground diameters were cultivated on a sterilized substrate under 16 h light/8 h dark before treatment. The four types of culture media were prepared as follows: potato dextrose agar (PDA), potato dextrose broth (PDB), Sabouraud dextrose agar (SDA), and Murashige and Skoog (MS) media; all the media were added with 1.5% agar and autoclaved at 121 °C for 20 min.

### 2.2. Isolation and Identification of Trichoderma Species

*Trichoderma* species were isolated from the soil samples using the plate dilution method [19]. Species identification was performed via a combination of morphological observation and molecular biological techniques. Morphological characterization was conducted on the basis of colony morphology, conidiophore structure, and conidial morphology. For molecular identification, the fragments of the ITS, *tef1α*, and *rpb2* genes were amplified via PCR in a 25 μL reaction system. Each reaction mixture contained 12.5 μL of 2× Taq PCR Master Mix, 1.0 μL forward primer (10 μmol/L), 1.0 μL reverse primer (10 μmol/L), and 1.0 μL genomic DNA template; sterile double-distilled water was added to bring the final volume to 25 μL. The uniform thermal cycling program was set as follows: initial denaturation at 94 °C for 3 min, followed by 35 cycles of denaturation at 94 °C for 30 s, annealing at 55 °C for 30 s, and extension at 72 °C for 45 s. A final extension step at 72 °C for 10 min was performed to complete all the PCR amplifications. Specific experimental procedures were carried out with reference to the methods described by Zhou et al. and Dou et al. [20,21]. The sequences of the three primer pairs used for gene amplification are provided in Supplementary Table S1, and all the obtained sequence data have been submitted to the National Center for Biotechnology Information (NCBI) database. Multiple sequence alignment was followed by phylogenetic reconstruction using maximum likelihood in MEGA X v.10.2.6 (https://www.megasoftware.net/). Bootstrap supports (1000 replicates) are labelled at nodes, and clades with values ≥70% were accepted as credible [22].

### 2.3. Selection of Superior Trichoderma Strains

#### 2.3.1. Determination of the Antifungal Activity of Single Strains

In this study, a total of 20 isolates belonging to three *Trichoderma* species were isolated. One strain with optimal growth performance was selected from each species for subsequent experiments, namely, *T. harzianum* (Tha354), *T. atroviride* (Tat368) and *T. asperelloides* (Tas369). Dual Culture Assay: Mycelial plugs (Φ = 5 mm) of *Trichoderma* and Cgl were separately placed on potato dextrose agar (PDA) plates at a linear distance of 6 cm for confrontation culture. Plates inoculated with only Cgl served as the control (Con). The radial mycelial growth of Cgl was measured once every 24 h by measuring two perpendicular colony diameters with a vernier calliper, and all recorded growth data are expressed in centimetres (cm). Fermentation Filtration Inhibition Assay: The *Trichoderma* isolates were cultured in PD liquid medium with shaking for 5 days, and then filtered through a 0.22 μm sterile filter membrane to obtain a sterile fermentation filtrate. The filtrate was mixed with molten PDA to prepare antifungal media with final concentrations of 50%, 33%, and 25% (*v*/*v*). Mycelial plugs of Cgl were inoculated onto these modified media, while PDA plates prepared with sterile water (instead of fermentation filtrate) and inoculated with Cgl served as the control. volatile organic compound (VOC) Inhibition Assay: A PDA plate inoculated with Cgl was inverted and placed on top of a PDA plate that had been preinoculated with *Trichoderma* and incubated for 48 h. The junction between the two plates was sealed with at least three layers of Parafilm to prevent volatile compounds from escaping. Plates inoculated with the pathogen alone served as the control. All antagonism tests involved incubation under a consistent light–dark cycle (12 h light/12 h dark). The colony diameter of Cgl was measured daily until the control group colonies covered most of the plate. Two perpendicular cross lines were drawn through the center of each colony to measure the colony diameters along the two orthogonal directions, and the average value was calculated to reduce measurement errors. The inhibition rate (IR%) was calculated using the following formula:

IR% = [(C − T)/C] × 100

where C represents the colony diameter of Cgl in the control group and T represents the colony diameter of Cgl in the treatment group. Each treatment was performed with three biological replicates. All strain abbreviations used in this manuscript are internal preservation codes for our laboratory’s indigenous *Trichoderma* resources.

#### 2.3.2. Antifungal Determination of Composite Strains

Strains Tas369, Tha354, and Tat368 were separately cultured on PDA medium and incubated at 28 °C under continuous light for 5 days. Spore suspensions were prepared by washing the cultures with sterile water. The spore concentration of each *Trichoderma* suspension was quantified using a hemocytometer and adjusted to 1 × 10^7^ spores/mL with sterile water. The three standardized suspensions were mixed at a 1:1:1 volume ratio to prepare the composite inoculant designated Tcom, with an overall spore concentration of 1 × 10^7^ spores/mL (one-third propagules from each strain); Mycelia of Cgl were inoculated into PD medium and incubated at 28 °C with shaking at 180 rpm under 12 h light/12 h dark photoperiod for 5 days. The culture was filtered through sterile gauze to collect the spore suspension. Spore suspensions was measured using a hemocytometer, and the suspension was adjusted with sterile water to a final concentration of 1 × 10^4^ spores/mL. A “soil-residue” microcosm system was established to simulate early spring conditions, aiming to evaluate the inhibitory effect of low-temperature *Trichoderma* early on the colonization of soil-borne Cgl pathogens. Surface soil samples were collected from the poplar forests and sterilized at 121 °C for 30 min, after which 150 g of sterilized soil was added to each sterile culture pot. The soil moisture content was adjusted to 60% of the field water-holding capacity, and 5 g of sterilized poplar leaves was evenly spread on the soil surface. An equal volume of Cgl spore suspension (1 × 10^4^ spores/mL) was uniformly sprayed onto the leaf layer. Five days later, the same area was sprayed with a Tcom spore suspension (1 × 10^7^ spores/mL). Three biological replicates were established for each treatment, and a control group without *Trichoderma* inoculation was included. The microcosm systems were cultured under controlled conditions: 12 h light/12 h dark photoperiod, 85% relative humidity, and 15 °C, with constant humidity maintained for 10 days. After cultivation, mixed soil-dead leaf samples were collected, and total genomic DNA was extracted using the cetyltrimethylammonium bromide (CTAB) method. Quantitative real-time PCR (RT–qPCR) was performed to determine the nucleic acid copy number of Cgl using pathogen-specific primers (listed in Supplementary Table S1).

#### 2.3.3. Screening of Plant Growth-Promoting Strains

Candidate disease-resistant *Trichoderma* strains were prepared into a spore suspension with a concentration of 1 × 10^7^ spores/mL, with an equal volume (100 mL) applied to the root zone of each seedling and seedlings receiving identical volumes of sterile water as controls. On days 7, 14, and 30 post-treatment, poplar seedlings were carefully uprooted from the substrate. After the plants were rinsed with clean water and gently blotted to remove surface moisture, the fresh weights of the aboveground shoots and underground roots were immediately determined using an electronic balance. Seedling height and total root length were measured with a standard ruler. Additionally, the stomatal density of newly expanded leaves was quantified under a Zeiss light microscope (40× objective, Carl Zeiss Microscopy GmbH, Jena, Germany), with at least 10 random fields examined per treatment.

### 2.4. Analysis of the Biological Control Mechanism of Dominant Strains

#### 2.4.1. Pathogen Challenge Assay and Physiological Biochemical Index Measurement

For the poplar seedling pathogenicity challenge, puncture inoculation was performed using a sterile needle to create uniform, tiny wounds on the leaves. An equal volume of Cgl spore suspension was then added onto each wound site for infection. To assess the control effect of each *Trichoderma* strain, the respective spore suspension (1 × 10^7^ spores/mL) prepared in Section 2.3.2 was separately sprayed onto poplar leaves. After 48 h of high-humidity incubation, leaves were mechanically wounded and then inoculated with mycelial plugs of Cgl. and incubated for another 7 days. Daily photographs were taken to monitor disease progression, and lesion areas were quantified. To evaluate the biocontrol efficacy of the dominant *Trichoderma* strains (Tcom and Tas369), a poplar seedling–anthracnose pathogen interaction system was established. Each uniform PdPap seedling received 100 mL of a *Trichoderma* spore suspension (1 × 10^7^ spores/mL) via root irrigation, and only single-root pretreatment with *Trichoderma* was performed in this experiment. After 48 h of *Trichoderma* pretreatment, all the seedlings were sprayed evenly with an equal volume of Cgl spore suspension (1 × 10^4^ spores/mL) and then incubated at 25 °C and 90% relative humidity for pathogen infection. The entire trial was carried out under a 12 h light/12 h dark photoperiod in a greenhouse, with three independent biological replicates containing 12 seedlings per replicate. Leaf samples were collected at 1 d, 3 d, 5 d, and 7 d post-Cgl inoculation. The activities of PAL and POD, along with the malondialdehyde (MDA) content, were determined using commercial assay kits (PAL: Cat. No. G0114F; POD: Cat. No. G0107F; MDA: Cat. No. G0109F) purchased from Suzhou Grace Biotechnology Co., Ltd. (Suzhou, China), to comprehensively evaluate the immune-inducing effect of *Trichoderma* on poplar seedlings and the degree of physiological damage to them after infection by Cgl. Three biological replicates were established for each time point.

#### 2.4.2. Quantification of Defense Gene Expression and Subepidermal Stomatal Observation

Quantitative real-time polymerase chain reaction (RT–qPCR) was used to characterize the expression dynamics of the key resistance signalling genes *NPR1* and five *PR* genes, with the PdPap Actin gene used as the internal reference. Gene expression was quantified via the 2^(−ΔΔCt)^ method; all values presented in the figures and the main text represent the fold change relative to the treatment solely inoculated with Cgl, without log_2_ transformation of the raw fold change data. Furthermore, nail polish blotting was used to prepare subepidermal impressions of leaves at key time points. Changes in the stomatal aperture and morphology of guard cells were observed under an optical microscope, with no fewer than 10 visual fields observed per treatment.

### 2.5. Statistical Analysis

All values are presented as the mean ± standard deviation (SD) of three independent biological replicates. Prior to parametric statistical testing, the normality of data residuals was assessed via the Shapiro–Wilk test, and homogeneity of variances was verified using Levene’s test; all datasets included in this study met the assumptions of parametric analysis. One-way analysis of variance (ANOVA) followed by Duncan’s multiple range test was employed to determine significant differences among groups using SPSS software (version 16.0; SPSS Inc., Chicago, IL, USA), with the significance threshold set at *p* < 0.05. Exact *p*-values for key pairwise comparisons are annotated in the Section 3 and corresponding figure legends.

## 3. Results

### 3.1. Isolation and Identification of Trichoderma

**Figure 1:**
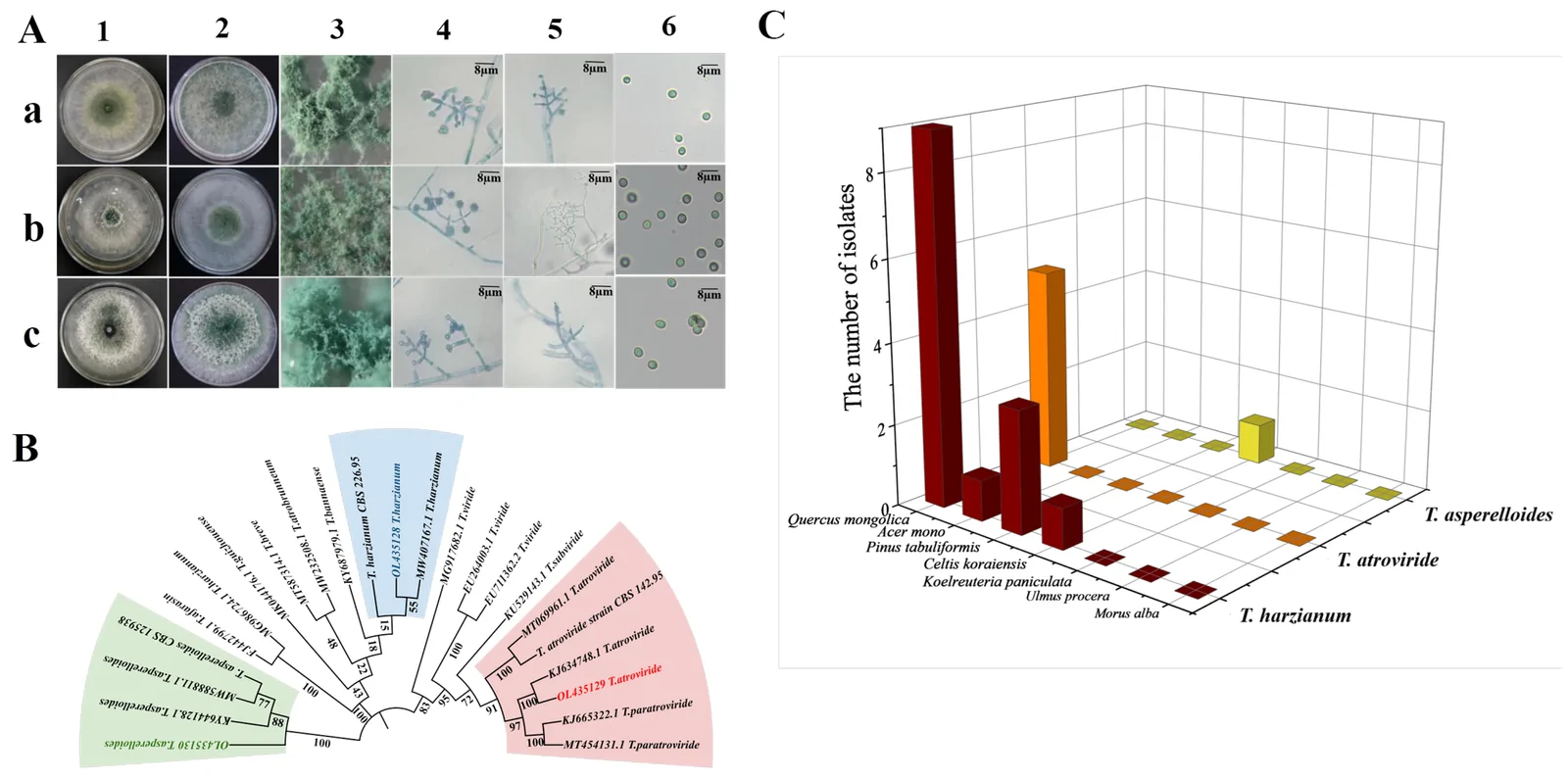
Isolation, identification and diversity analysis of *Trichoderma*. (**A**) Microscopic morphological characteristics of *T. harzianum* (a), *T. atroviride* (b), and *T. asperelloides* (c). 1 PDA medium, 2 SDA medium, 3 Conidiogenous clusters, 4 conidiophores, 5 Phialide, and 6 conidia. (**B**) Phylogenetic analysis of three *Trichoderma* species using the *tef1α* gene. (**C**) Quantity and species of *Trichoderma* in soil around tree roots.

A total of 20 *Trichoderma* strains were isolated from rhizosphere soil samples of seven tree species, namely *Quercus mongolica*, *Acer mono*, *Pinus tabuliformis*, *Celtis koraiensis*, *Koelreuteria paniculata*, *Ulmus procera*, and *Morus alba*, which were collected from Tianzhu Mountain, Shenyang City, Liaoning Province, China. These 20 *Trichoderma* strains were named NECC21350-21369 according to the laboratory’s preservation numbering system. On the basis of their morphological characteristics and ITS sequence alignment, the 20 isolates were preliminarily classified into three species: *T. harzianum*, *T. atroviride* and *T. asperelloides* (Figure 1A). Phylogenetic analysis was performed on *tef1α* and *rpb2* sequences of *Trichoderma* spp. with high bootstrap support. The results indicated that the *Trichoderma* isolates were clustered with the same reference sequence (Figure 1B and Figure S1). Sequences within the same clade were considered to be closely related. Finally, 14 *T. harzianum* isolates, 5 *T. atroviride* isolates and 1 *T. asperelloides* isolate were obtained.

The molecular identification sequences of the strains used in the later stage of the experiment were submitted to the National Center for Biotechnology Information (NCBI) database. Among these strains, NECC21354 was identified as *T. harzianum* (designated Tha354). The GenBank accession numbers for the ITS region, *tef1α* gene, and *rpb2* gene are OL377834, OL435125, and OL435128, respectively. NECC21368 was identified as *T. atroviride* (designated as Tat368). The corresponding GenBank accession numbers for the abovementioned three molecular markers are OL377848, OL435126, and OL435129. NECC21369 was identified as *T. asperelloides* (designated Tas369). The GenBank accession numbers for the abovementioned three molecular markers are OL377849, OL435127, and OL435130. Diversity analysis revealed that the number of *Trichoderma* isolated from the rhizosphere of *Q. mongolica* was the greatest (14 strains), followed by that from *P. tabuliformis* (3 strains). *T. harzianum* was the dominant species in this region, accounting for 70% of the total isolated strains (Figure 1C). Collectively, three representative isolates, Tha354, Tat368 and Tas369, covering all three detected species, were selected for subsequent biocontrol functional assays.

### 3.2. Evaluation of the Antifungal Capacity of Trichoderma

**Figure 2:**
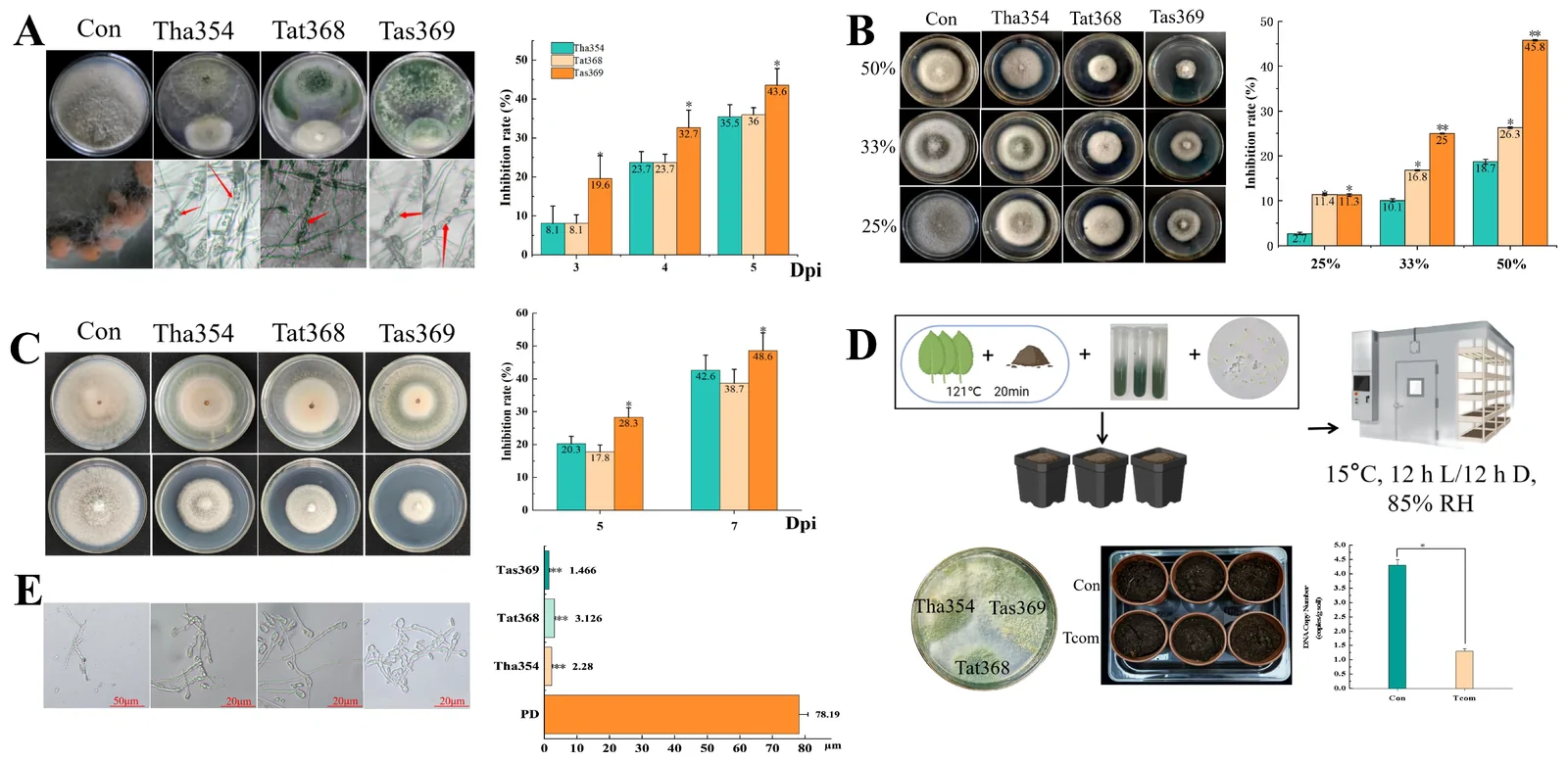
Direct antifungal effect determination of three *Trichoderma* strains. The plates show the state after 7 days of cultivation. (**A**) Antagonism experiment between *Trichoderma* and Cgl, with the upper part showing *Trichoderma* and the lower part showing Cgl and the inhibition rate of *Trichoderma* on Cgl. The arrow points to the phenomenon of mycoparasitism of Cgl by *Trichoderma*. (**B**) Inhibitory effects and inhibition rates of the culture filtrates from 3 dominant *Trichoderma* strains on anthracnose fungus growth. (**C**) Inhibitory effects and inhibition rates of volatile compounds from three dominant *Trichoderma* strains on anthracnose fungal growth. (**D**) Detection of the inhibitory effects of *Trichoderma* on spring spore germination in a soil environment simulation. (**E**) Morphology and length of germ tubes after 24 h of treatment with Cgl spores with culture filtrates from 3 dominant *Trichoderma* strains. Values marked with * indicate significant differences at *p* < 0.05, and ** represents highly significant differences at *p* < 0.01.

To evaluate the biocontrol potential of *Trichoderma* species against Cgl and screen out dominant strains, three complementary methods were used in this study: plate confrontation culture, culture filtrate testing, and VOC assessment. In plate confrontation assays, strain Tas369 displayed the strongest antifungal activity, with a 43.6% inhibition rate, completely overgrowing the pathogen colonies and producing abundant spores. Strain Tat368 formed distinct inhibitory zones around its colonies. Furthermore, the mycelial growth inhibition rates of Tha354 and Tat369 against *C. gloeosporioides* (Cgl) reached 35.5% and 36.0%, respectively (Figure 2A). At the interaction interface, microscopic observation showed that the hyphae of the three *Trichoderma* strains formed tight coils around Cgl hyphae, causing growth inhibition and mycelial disintegration at the sites of contact. The nonvolatile metabolites of the three strains inhibited Cgl growth at different concentrations, significantly disrupting mycelial branching and resulting in sparse colonies, and Tas369 had the strongest inhibitory effect (45.8%) (Figure 2B). After 7 days of coculture, the VOCs produced by these *Trichoderma* strains inhibited Cgl growth by 48.5% (Figure 2C). In addition, the fermentation filtrates of the *Trichoderma* strains inhibited Cgl spore germination by more than 97% (Figure 2E). Simulated spring spore germination tests indicated that adding Tcom for 10 days reduced the survival rate of Cgl by nearly 70% (Figure 2D). Overall, Tas369 achieved 43.6% mycelial inhibition in dual culture, 45.8% inhibition via nonvolatile metabolites, and 48.5% inhibition from VOCs, exhibiting the strongest overall antagonistic activity across all three in vitro assays and demonstrating comprehensive, prominent antifungal effects through diverse biocontrol mechanisms, indicating that it is the dominant strain for biocontrol against Cgl.

### 3.3. Pathogenicity Assay of Cgl and Direct Biocontrol Efficacy of Trichoderma Against Anthracnose

**Figure 3:**
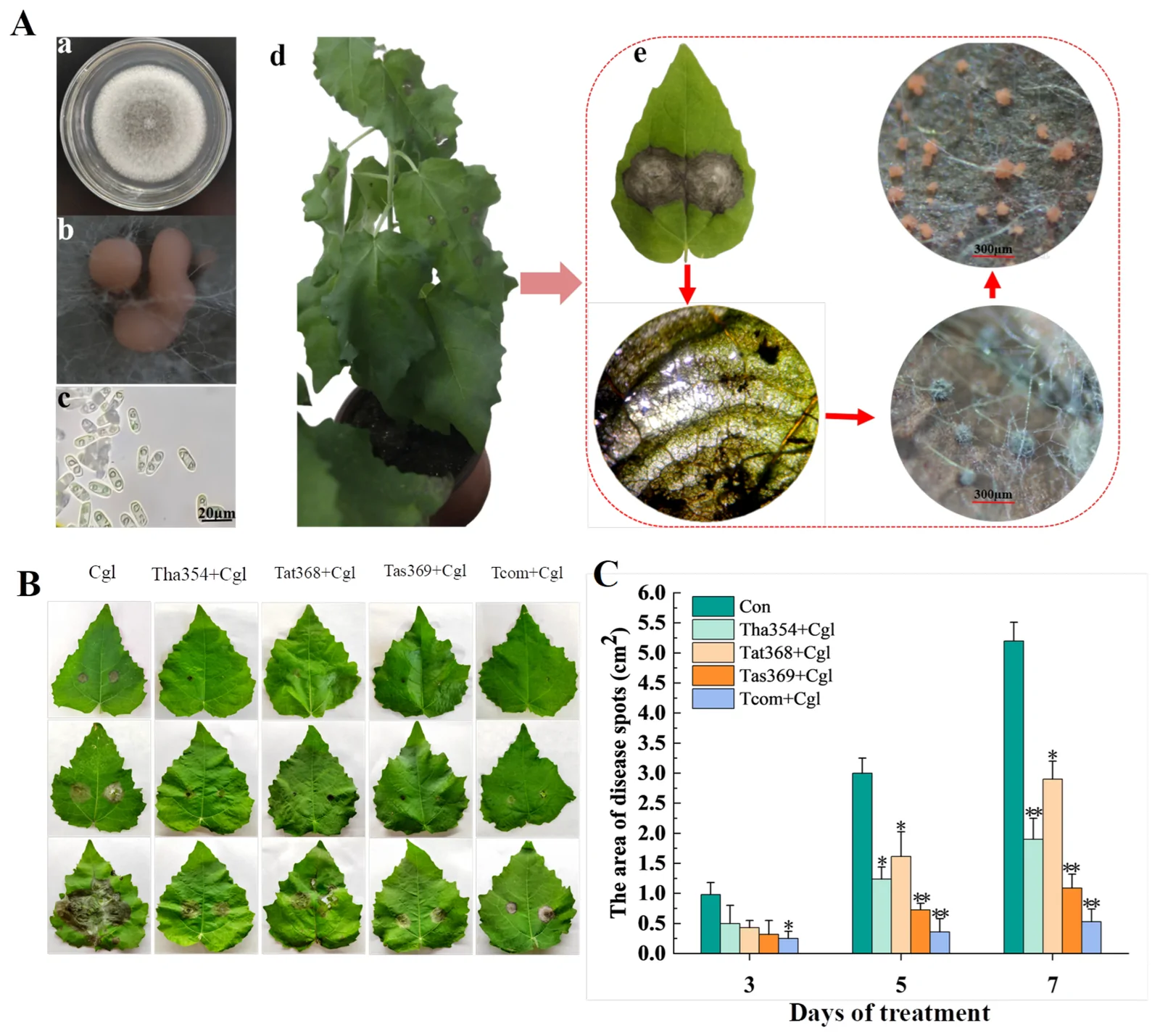
Screening of dominant *Trichoderma* strains for direct inhibition of Cgl. (**A**) Microscopic observation and pathogenicity test of Cgl. (**a**) Colony growth, (**b**) Sporodochia morphology and (**c**) conidial morphology, (**d**,**e**) Phenotype of PdPap 7 days after infection with Cgl. (**B**,**C**) Observation and quantification of lesion areas at 3, 5, and 7 days after Cgl inoculation. Values marked with * indicate significant differences at *p* < 0.05, and ** represents highly significant differences at *p* < 0.01.

To clarify the pathogenic damage caused by Cgl to poplar leaves and the ability of *Trichoderma* to control disease spread, this study carried out inoculation and biological control experiments. After the poplar leaves were punctured and inoculated with Cgl, necrotic lesions first emerged on the leaf surface; 7 days post-inoculation, the lesions further expanded into large ring-shaped lesions. In the late stage of the disease, blue–gray conidial discs with setae formed at the lesion sites, and the conidia mixed with the mucus were extruded from the discs, resulting in the formation of orange–red curled conidial horns (Figure 3A). These typical symptoms indicate that Cgl can cause severe damage to poplar leaves, highlighting the necessity of timely implementation of effective disease control measures. The results of a control experiment involving the foliar spraying of a *Trichoderma* spore suspension demonstrated that the three tested single *Trichoderma* strains and their combined treatments could reduce disease severity to varying degrees. Seven days after treatment, the control efficacy of the *Trichoderma* strains against leaf disease ranged from 46% to 90%. Significant differences in control efficacy were observed among the strains: the Tcom spraying treatment exhibited the greatest efficacy (89.8%), followed by Tas369 (79.0%), while Tat368 showed relatively poor control efficacy (Figure 3B,C).

### 3.4. Screening of Superior Growth-Promoting Strains

**Figure 4:**
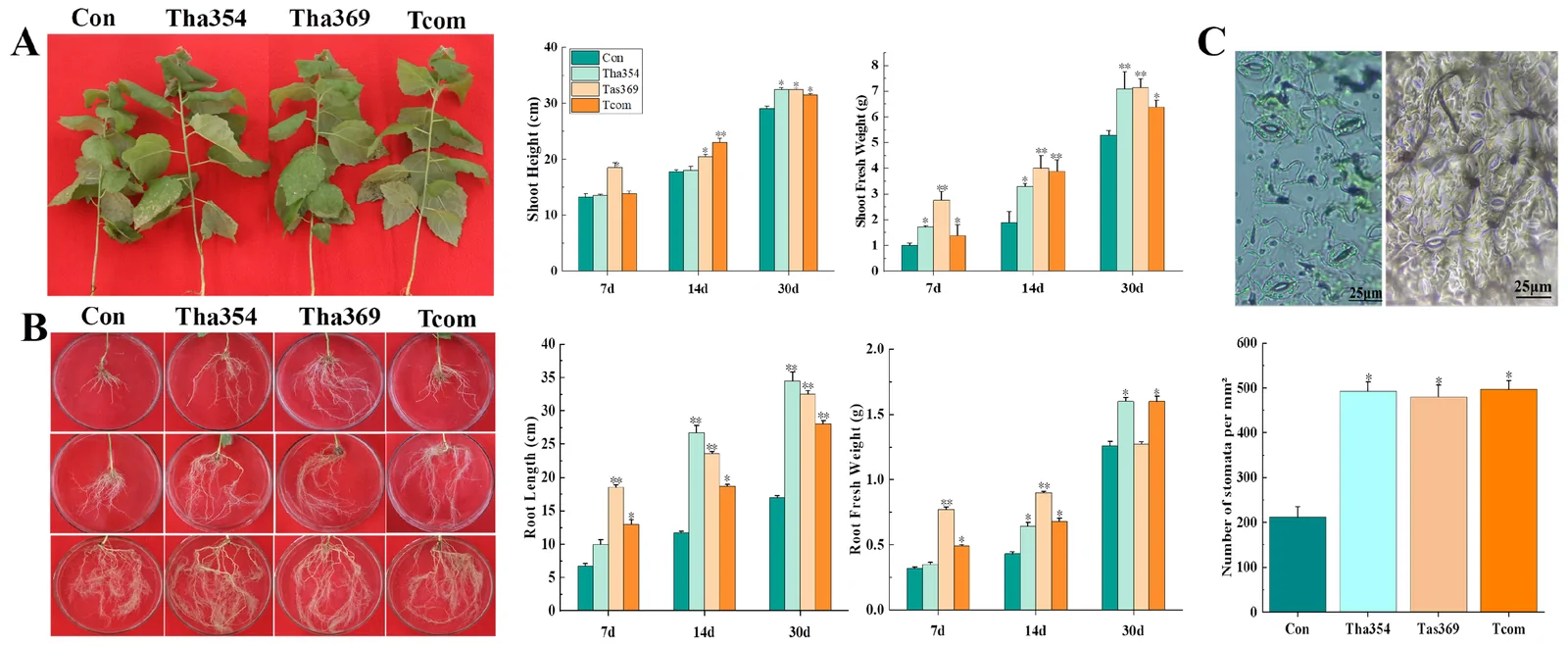
Effects of *Trichoderma* spore suspension on the growth of PdPap seedlings. (**A**) Morphology of the aboveground parts of poplar plants after 30 days of *Trichoderma* treatment and measurements of shoot height and fresh weight at 7 d, 14 d, and 30 d. (**B**) Morphology of the underground parts of poplar plants after 30 days of *Trichoderma* treatment and measurements of root length and fresh weight at 7 d, 14 d, and 30 d. (**C**) Stomatal morphology and density on the undersides of newly emerged leaves in poplar plants 30 d after treatment. Values marked with * indicate significant differences at *p* < 0.05, and ** represents highly significant differences at *p* < 0.01.

Based on the disease resistance screening results described above, we further evaluated the growth-promoting effects of the dominant *Trichoderma* strains on poplar seedlings, aiming to identify superior strains with both biocontrol and growth-promoting functions. Both single-strain and mixed treatments enhanced the growth of shoots and roots (Figure 4). The Tcom treatment increased shoot fresh weight by 3.63-fold (Figure 4A). During the 7–14-day treatment period, Tas369 more than doubled the fresh weights of both shoots and roots relative to the control, indicating that Tas369 rapidly promoted biomass accumulation and resulted in robust, disease-resistant plants. After 30 days, the Tha354 treatment increased root length 2.4-fold and fresh root weight 3.57-fold (Figure 4B); in addition, the highest stomatal density among all treatment groups reached 497 stomata/mm^2^, which was 2.34 times that of the control (Figure 4C). Collectively, these results demonstrate that Tas369 and Tcom possess outstanding antifungal and growth-promoting properties.

### 3.5. Biological Control Mechanism of Dominant Strains

**Figure 5:**
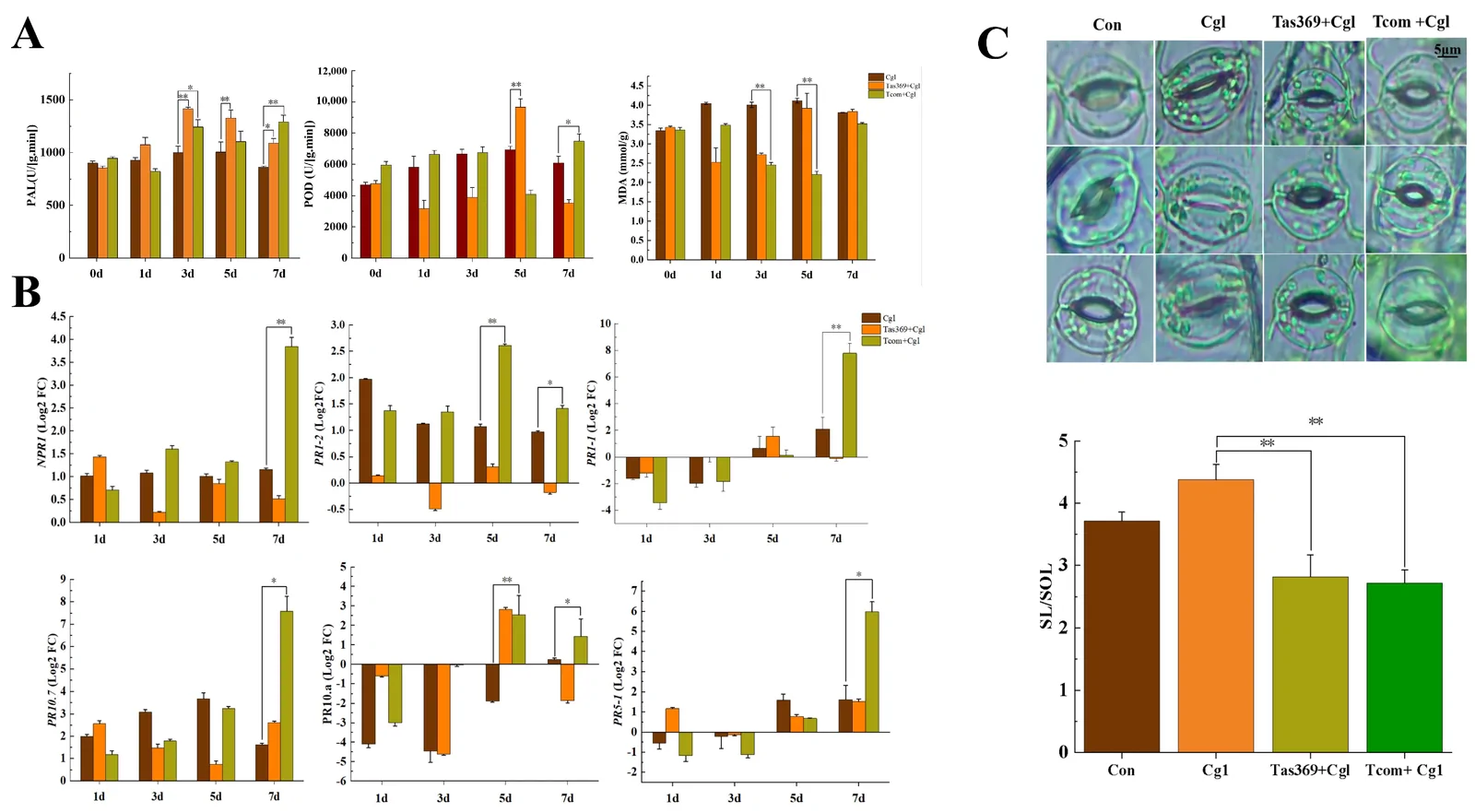
Biocontrol mechanisms of dominant *Trichoderma*. (**A**) Defense enzyme activities, from left to right: PAL, POD, and MDA. (**B**) Relative fold changes in the expression of six disease resistance-related genes (*NPR1*, *PR1-2*, *PR1-1*, *PR10.7*, *PR10.a*, and *PR5-1*) in poplar leaves under different treatments. All values represent fold changes calculated via the 2^(−ΔΔCt)^ method relative to the pathogen-only control. (**C**) Stomatal morphology and relative stomatal opening ratio (SL/SOL). SL: total length of guard cells; SOL: length of stomatal pore opening. Higher SL/SOL value indicates more closed stomata. Values marked with * indicate significant differences at *p* < 0.05, and ** represents highly significant differences at *p* < 0.01.

Based on the comprehensive screening results for antifungal and growth-promoting activities, we further investigated the biocontrol mechanisms of the dominant strains Tas369 and Tcom, with particular emphasis on their ability to induce disease resistance in poplar. This induction was systematically evaluated from three perspectives: physiological metabolism, gene expression, and leaf morphology. Physiologically, Tas369 significantly enhanced PAL activity in poplar, with a marked increase observed between days 3 and 5 post-treatment; the maximum increase reached 434.4 U/(g·min) relative to the control. On day 5, POD activity in the Tas369-treated group was 4070.4 U/(g·min) higher than that in the pathogen-only inoculated group. For Tcom, a notable reduction in MDA content occurred from day 3 to day 5, with the largest decrease amounting to 1.1 mmol/g (Figure 5A). In addition, compared with the control, Tcom markedly elevated PAL activity, which was 427 U/(g·min) higher on day 7. Quantitative transcriptional analysis revealed that after 7 days of Tcom treatment, the expression levels of key defense-related genes—*NPR1* (14.32-fold), *PR1-1* (219.79-fold), *PR5-1* (62.68-fold), and *PR10.7* (191.34-fold)—were significantly upregulated relative to the control (Figure 5B).

Morphological observation revealed that, in contrast to the control group, guard cells in the *Trichoderma*-treated groups (Tas369 and Tcom) gradually regained turgor, with stomatal morphology restored and aspect ratios consistently ranging from 2.7 to 2.9. These results suggest that *Trichoderma* promotes disease resistance in poplar by alleviating the stomatal dysfunction caused by pathogen infection. In contrast, stomata on the abaxial leaf surface of the pathogen-only inoculated group remained nearly closed to slightly open, with apertures maintained at 50–60% of the control level (Figure 5C), indicating successful pathogen invasion and impairment of the plant’s innate immune response.

## 4. Discussion

### 4.1. Native Trichoderma Community Characteristics and Strain Screening Value

Our strain isolation survey of seven tree rhizospheres on Tianzhu Mountain revealed pronounced host-driven differentiation of local *Trichoderma* populations (Figure 1). The *Q. mongolica* rhizosphere contained the greatest number of isolates (14 strains), with *T. harzianum* as the dominant species accounting for 70% of the total isolates. This pattern aligns with the known latitudinal distribution of *Trichoderma* communities in China: cold-temperate northern forest soils are colonized mainly by the *T. harzianum* species complex and *T. atroviride*, while *T. asperelloides* (Tas369) has been reported to be recovered in Liaoning forests [11,23,24]. In contrast, the prevalence of dominant species in southern regions has shifted to warm–humid-adapted taxa such as *T. viride*, while the prevalence of *T. harzianum* has decreased [25]. At the microhabitat scale, root exudates drive pronounced differences in *Trichoderma* communities among forest tree rhizospheres even within the same habitat. In Heilongjiang, Juglans mandshurica and *Pinus koraiensis* rhizospheres are dominated by *T. pseudoharzianum*, *Quercus mongolica* by *T. virens*, and *Acer saccharum* exclusively by *T. atroviride* [18]. Critically, the local strains obtained herein exhibit strong low-temperature tolerance and retain stable antagonistic activity at 15 °C, which strongly supports the consensus that indigenous isolates exhibit more reliable field biocontrol performance than exotic strains do. For future forest biocontrol agent exploitation, targeted rhizosphere sampling from native tree species is an efficient strategy for obtaining functional *Trichoderma* resources for microbial fertilizer development.

### 4.2. Multipathway Antagonism and Litter-Based Preemptive Biocontrol of Trichoderma

*Trichoderma* and its metabolites effectively antagonize multiple forest pathogenic fungi. Poletto et al. reported that various *Trichoderma* species directly inhibit hickory anthracnose pathogens. Specifically, *T. virens* and *T. tomentosum* strongly suppress *C. gloeosporioides* growth via mycoparasitism, with inhibition rates reaching 40.8% [26]. Herein, three *Trichoderma* strains exerted competitive effects on pathogenic Cgl for nutritional resources and ecological niches, their hyphae tightly interwove, yielding a maximum inhibition rate of 48.5%, which aligns with prior research findings. Additionally, *T. koningiopsis* T2 synergistically suppresses *C. gloeosporioides* and *Alternaria alternata* via mycoparasitism, VOCs, and nonvolatile metabolites [27]. Similarly, our findings demonstrate that the antifungal activity of Tas369 against Cgl is mediated by these three mechanisms, with a maximal inhibition rate of 48.5%. Furthermore, its metabolite filtrate diminished Cgl colony density and repressed spore germination, with the latter reaching an inhibition rate of up to 97%, indicating that the metabolites exhibit a targeting bias toward the vegetative growth phase of Cgl.

The antifungal activity of *Trichoderma* also extends to the preemergence field control of early-season anthracnose. Following overwintering, latent hyphae of *Colletotrichum* in diseased plant debris reinitiate metabolic activity at 10–15 °C—a temperature regime that coincides with the early leaf flush stage of poplar [28]. This phenological overlap enables the direct application of *Trichoderma* to soil-surface plant litter for proactive biocontrol, thereby suppressing pathogen germination and subsequent dispersal in the field. In vitro simulations of spring pathogen germination conditions in our study revealed a maximum inhibition rate of ~70% (Figure 2D). These results provide empirical evidence that the application of *Trichoderma* to leaf litter can achieve early preventive control by competing for substrate nutrients, parasitizing residual pathogen hyphae and releasing antifungal metabolites [29,30,31]. Together, our in vitro and simulated field data prove that *Trichoderma* can construct a multilayered biocontrol system capable of suppressing pathogen growth both in pure culture and on overwintering plant debris.

### 4.3. Trichoderma Coregulates Poplar Growth and Stomatal Traits

Our pot trial demonstrated that *Trichoderma* treatments significantly increased poplar aboveground and underground biomass, accompanied by a 134% increase in stomatal density of newly expanded leaves (reaching 497 stomata/mm^2^; Figure 4C), along with restored normal stomatal morphology and aperture (Figure 5C). These structural and functional changes collectively enhanced photosynthetic potential, contributing to integrated growth promotion and disease resistance benefits. While most prior research on *Trichoderma*-mediated growth promotion in woody plants has focused exclusively on seedling height and root architecture [32,33], the regulation of stomatal traits in trees remains poorly documented, with existing stomatal-related studies largely confined to crops—for instance, *T. asperellum* reduced stomatal number but increased total aperture in cocoa, and enhanced stomatal density by 44.2% in tomato [34,35]. Our work extends this research to poplar, revealing a novel physiological regulatory pathway unique to forest trees and addressing the research gap noted in the Introduction concerning the limited understanding of *Trichoderma*-mediated physiological modulation in woody hosts.

Our findings reveal that *Trichoderma* simultaneously modulates macroscopic plant morphology and microscopic leaf epidermal structures, offering multidimensional evidence for microbe–plant growth adaptation mechanisms.

### 4.4. Tcom Strongly Activates SA/JA Systemic Defense Signalling and Repairs Pathogen-Disrupted Stomatal Function

Furthermore, compared with those in the pathogen-only group, the transcript levels of six defense-related genes in the *Trichoderma*-treated group were significantly greater. These findings indicate that *Trichoderma* activates SA/JA signalling pathways. Notably, *NPR1* and *PR1* upregulation (up to 219.79-fold) confirmed that *Trichoderma* successfully triggered SAR in plants, resulting in a long-term “prevention–defense” effect rather than a short-term passive stress response. In poplar–black spot disease interactions, *T. asperellum* significantly increases *PR1*, *PR5* (5–6-fold increase), and *PR10* (3–5-fold increase) expression [9], which is consistent with our findings of *Trichoderma*-induced defense gene upregulation, corroborating the generality of this defense activation mechanism in poplar disease control.

Stomatal homeostasis represents an additional defensive layer regulated by *Trichoderma*. Prior investigations have shown that treatment of *Arabidopsis thaliana* with *T. virens* G-41 significantly delayed stomatal closure induced by *Pseudomonas syringae* pv. tomato DC3000, thereby sustaining the normal stomatal opening–closing rhythm [36]. Similarly, our observations revealed that the application of *Trichoderma* alleviated the abnormal stomatal movement triggered by *C. gloeosporioides* and restored normal stomatal morphology in poplar leaves, which may have contributed to the combined benefits of disease resistance and improved seedling growth.

In conclusion, this study illustrates that the composite agent *Trichoderma* alleviates poplar anthracnose caused by Cgl via two coordinated beneficial effects: triggering plant defense responses and restoring regular stomatal opening and closing patterns. This work provides preliminary experimental support for the application of compound *Trichoderma* inoculants in forest seedling disease management and for overcoming the functional deficiency of single-strain biocontrol agents.

### 4.5. Advantages of Compound Trichoderma Inoculants over Single Strains

Recent investigations have increasingly demonstrated that the integration of *Trichoderma* strains with distinct functional traits elicits remarkable synergistic effects in agroforestry ecosystems, thereby surmounting the inherent limitations associated with single-strain applications. Han et al. reported that composite *Trichoderma* treatments not only induced peak expression of *PR1*, *PR5*, *PR10*, and *MYC-R* in poplar at 5 days post-inoculation with *Nigrospora oryzae* (with a maximum fold change of 17.32 relative to the control) but also significantly increased aboveground biomass, plant height (both exceeding 1.7-fold compared with the control), and root growth performance [10]. Similarly, Kumar et al. reported that compared with single-strain treatments, the coapplication of *T. erinaceum* NAIMCC-F-02171 and *T. viride* NAIMCC-F-02500 substantially increased the growth parameters (leaf weight, stem length, and root weight) of bean plants infected with *Sclerotinia sclerotiorum* while concurrently increasing the activities of multiple antioxidant enzymes [37]. In this study, the application of a mixture of three dominant *Trichoderma* strains significantly increased the stomatal density in newly emerged poplar leaves (2.34-fold relative to that of the control) and potently induced systemic resistance against anthracnose. Specifically, PAL activity reached 427 U/(g·min), while the expression levels of *PR1-1* and *PR10.7* were upregulated to 219.79-fold and 191.34-fold that of the control, respectively. Collectively, these findings underscore the comprehensive advantages of composite strains in integrating biocontrol and growth-promoting functions, which are superior to those of single-strain applications.

We note that the current experimental design cannot statistically distinguish true synergism from additive effects or dominant single-strain activity, nor can it rule out the possibility that improved efficacy originates from increased total *Trichoderma* spore concentration. The superior comprehensive performance of Tcom is therefore consistent with potential synergistic interactions among the three isolates. Formal verification of strain synergism via Bliss independence, Loewe additivity, or full factorial models will be conducted in dedicated follow-up trials. Even so, our results clearly illustrate that composite *Trichoderma* inoculants outperform single strains in terms of pathogen suppression, oxidative damage alleviation, immune induction and seedling growth promotion.

In conclusion, future development of *Trichoderma* resources should adopt an integrated collection strategy to acquire functionally diverse strains. Furthermore, an increasing number of studies have confirmed that *Trichoderma* species can establish multilevel biocontrol systems and coordinately modulate plant immunity and stomatal behavior, indicating that these plants have substantial potential for application in green disease management practices. While the results of the present study verify that *Trichoderma* significantly increases stomatal density in poplar, the underlying molecular regulatory network remains elusive—for instance, whether SA/JA signalling directly modulates the expression of stomatal development-related genes (e.g., *SPCH* and *MUTE*) and whether elevated stomatal density is causally associated with enhanced disease resistance and growth promotion warrant further verification. Thus, in-depth elucidation of the molecular mechanisms governing *Trichoderma*–plant interactions, particularly the regulatory pathways of stomatal density, will emerge as a pivotal research direction for unlocking and augmenting its practical efficacy in agricultural and forestry systems.

## 5. Conclusions

**Figure 6:**
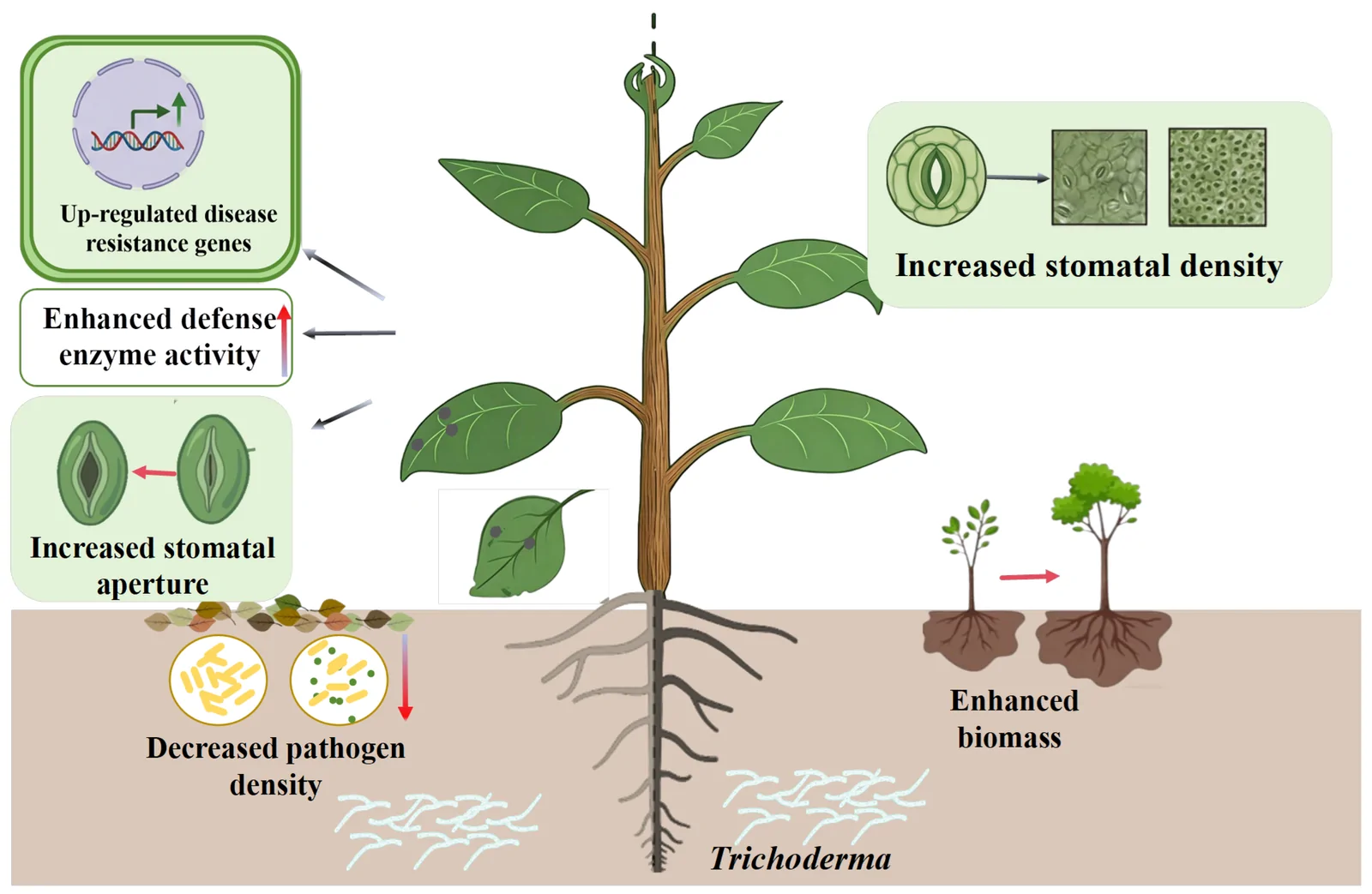
Biocontrol mechanism of *Trichoderma*-mediated protection of poplar against Cgl. The gradient arrow in the figure indicates the same trend as described in the text.

In this study, three dominant *Trichoderma* strains (Tha354, Tat368 and Tas369) were isolated from the rhizosphere of forest trees on Tianzhu Mountain, Shenyang. Both *Trichoderma* *asperelloides* (Tas369) and the consortium of the three isolates (Tcom) demonstrated excellent dual functionality, exhibiting remarkable biocontrol efficacy against poplar anthracnose while promoting poplar growth. Tas369 effectively inhibited the pathogen causing poplar anthracnose through multiple mechanisms, whereas Tcom significantly reduced the survival rate of overwintering pathogens under low-temperature conditions, achieving a biocontrol efficacy of 89.8% when applied as a foliar treatment. Both strains significantly increased the leaf biomass and stomatal density in newly developed poplar leaves, upregulated the expression of disease resistance-related genes, activated defense signalling pathways, and restored stomatal morphology and function. Notably, compared with each individual strain, Tcom displayed better overall performance, and this superior phenotype is consistent with potential synergistic interactions among the three isolates. Tas369 represents an ideal candidate for single-strain biocontrol applications, whereas Tcom exhibited superior overall performance. Collectively, these strains offer valuable microbial resources and technical support for the green management of poplar anthracnose. The mechanisms underlying the biocontrol and growth-promoting effects of *Trichoderma* on poplar resistance to *C. gloeosporioides* (Cgl) are described in Figure 6. This study offers theoretical and experimental evidence for the biocontrol of poplar anthracnose caused by Cgl.

## Data Availability

The original contributions presented in this study are included in the article/Supplementary Materials. Further inquiries can be directed to the corresponding author.

## Supplementary Materials

The following supporting information can be downloaded at https://www.mdpi.com/article/10.3390/microorganisms14081817/s1. Table S1: Genes and Sequences of Primers used in this study; Figure S1: Phylogenetic analysis of three *Trichoderma* species based on the *rpb2* gene.
