# Supplementary material for: Mechanisms of Biocontrol on Poplar Anthracnose and Growth Promotion by Trichoderma asperelloides Tas369 and Tri-Trichoderma Composite Strain
Source: Microorganisms. 2026 Aug 18;14(8):1817. doi: 10.3390/microorganisms14081817 (PMC13515690; doi:10.3390/microorganisms14081817)
Supplement: Supplementary file 1 [file microorganisms-14-01817-s001.zip › microorganisms-4420516-supplementary.pdf]

Table S1. Genes and Primer Sequences Used in This Study

Gene	primer	5′--3′
ITS	ITS5	TCCTCCGCTTATTGATATGC
	ITS4	GGAAGTAAAAGTCGTAACAAGG
tef1- α	EF1-281F	CATCGAGAAGTTCGAGAAGG
	TEfillerev	AACTTGCAGGCAATGTGG
rpb2	FRPB2-5F	GA(T/C)GA(T/C)(A/C)G(A/T)GATCA(T/C)TT(T/C)GG
	FRPB2-7Cr	CCCAT(A/G)GCTTG(T/C)TT(A/G)CCCAT
actin	actin-L	TTCCGTTGCCCTGAGGTCCTAT
	actin-R	TCAGGAGGAGCAACCACCTTGA
PR1-2	PR1-2L	CCTCCACCGCAACTCACTTCTT
	PR1-2R	CCAGCAGTGAAGAGGGAAACGA
PR10a	PR10a-L	GGTCAATGAGGAGGAGATAGACGC
	PR10a-R	TCACTGCCCACGCTTTCCAAA
NPR1	NPR-L	GGCCGACGATACTTCCCTAGTT
	NPR-R	TGCCCTCATAGTTTCCTGAGCT
PR10.7	PR10.7L	CGAATCGTGTCCGTCAGAGCTT
	PR10.7R	TCGGTGGATGCACAAGTGAAGG
PR1-1	PR1-1L	GGCAAACCGATTCTTGGCTCCT
	PR1-1R	ACATTTACGCTCCGAGACCCAC
PR5-1	PR5-1L	TTCACTTGTGCCTCCGCTGATT
	PR5-1R	GCTGACAATTCCCGCAATGACA
Cgl-actin	Cgl-actin-L	GTATCGTCAAGAAGTGGGAGG
	Cgl-actin-R	TGAGACCTTGGGCGTAGAGT
Cgl-CgE3	Cgl-CgE3-L	ATGGTGATGGTGCAACGGT
	Cgl-CgE3-R	TGCTCCTCCTGCTCCTCGT

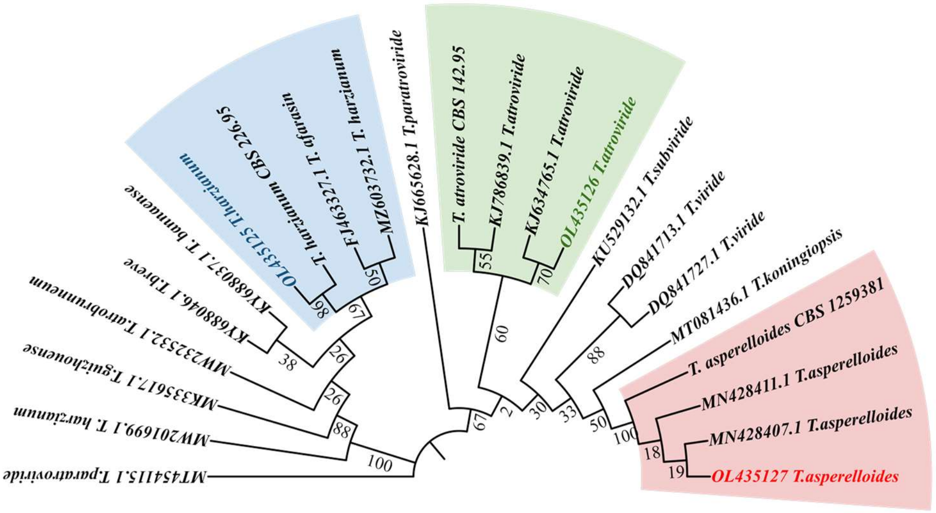

Figure S1. Phylogenetic analysis of three *Trichoderma* species based on the *rpb2* gene
